# Effects of physical health beliefs on college students’ physical exercise behavior intention: mediating effects of exercise imagery

**DOI:** 10.1186/s40359-024-01558-3

**Published:** 2024-02-26

**Authors:** Li Zhang, Donghuan Bai, Pengwei Song, Jia Zhang

**Affiliations:** 1 School of Physical Education, Chongqing University, Chongqing, China; 2 School of Physical Education, Huaibei Normal University, Huaibei, China; 3 School of Physical Education, Guangxi Science and Technology Normal University, Laibin, China

**Keywords:** Physical health beliefs, Exercise imagery, Physical exercise behavior intention, College student, Mediating effects

## Abstract

**Objective:**

This study explores the relationship between physical health beliefs and physical exercise behavior intention of college students and constructs a mediation model through the mediation role of exercise imagery.

**Methods:**

Using the stratified cluster sampling method, 1356 college students were measured in group psychology by using the Physical Health Beliefs Scale, Exercise Imagery Inventory, and Physical Exercise Behavior Intention Scale. The statistical software Mplus 8.1, SPSS 22.0 and SPSS PROCESS 3.3 were used for statistical processing. The common method deviation test was carried out by Harman single-factor control method. Finally, the bootstrap sampling test method and process plug-in were used to test the significance of intermediary effect.

**Results:**

(1) physical health beliefs have a significant predictive effect on physical exercise behavior intention (β = 0.32, *p* < 0.001); (2) exercise imagery (β = 0.13, *p* < 0.001) mediate the relationship between physical health beliefs and physical exercise behavior intention (physical health beliefs → exercise imagery → physical exercise behavior intention (95% Cl: 0.14, 0.32)).

**Conclusion:**

physical health beliefs can directly improve the physical exercise behavior intention of college students, which can also affect college students’ physical exercise behavior intention indirectly through exercise imagery. The findings suggest that exercise imagery are important variables that mediate the effect of the college students’ physical health beliefs on their physical exercise behavior intention.

## Introduction

The survey data in 2021 showed that the obesity rate and myopia rate of college students remain high, while depression and anxiety caused by psychological problems occur frequently [1]. In this context, effectively improving college students’ health awareness and cultivating active health behaviors are needed in today’s college physical education curriculum. The state, society, and schools are paying more and more attention to sports, and the promulgation of several national policies provides essential guarantees for sports development in China. Therefore, after experiencing the threat of many external environmental (COVID-19, influenza A) stimuli, popularizing sports health knowledge, improving sports health awareness, and enhancing physical health is a strong guarantee for constructing a healthy China.

Studies have shown that physical health significantly impact people’s physical and psychological stress [2]. In addition, the health belief model(HBM) posits that when an individual perceives that his/her health is threatened, he/she adopts healthy behavior by employing positive beliefs [3]. Based on motivation, cognitive, and value expectancy theory, HBM focuses on people’s beliefs about health and values the internal and external factors that influence beliefs [4, 5]. HBM is widely used to predict health risks, interpret health risk factors and modify health behaviors, providing a theoretical framework for behavioral change [6, 7]. In addition, HBM posits that when an individual perceives that his/her health is threatened, he/she adopts healthy behavior by employing positive beliefs [8]. HBM emphasizes the perception and behavioral assessment of the threat of disease [9]. When individuals perceive the seriousness of a disease threatening their physical health, it affects their expectation of the value of exercising, which partially reveals the realistic rationality of the stress process model [10, 11]. Studies have shown that when the perceived threat is high, this perception is a strong indicator of behavioral change [12].

On the other hand, within the exercise domain, the theory of planned behavior (TPB) has been applied to study determinants of exercise behavior [13–15]. According to this theory, exercise is determined primarily by the intention to exercise: the intention is considered to summarize a motivation to act, which is determined by attitudes, subjective norms, and perceived behavioral control. Physical exercise behavior intention refers to an individual’s psychological process of consciously and purposefully planning to participate in physical exercise [16]. It is the state of preparation before participating in a sport, not the action itself; it is the psychological tendency before the action. According to cognitive-behavioral theory, cognition coordinates behavior, directly affecting whether the individual finally takes action [17]. It has also been shown that physical health beliefs significantly impact exercise behavior [18]. Relevant research shows that the physical fitness of Chinese college students show a downward trend, the improvement of college students’ physical fitness is “imminent”, and it is very important to strengthen health education in the student population, enhance their awareness of exercise and encourage students to adhere to exercise.

In recent years, researchers have studied physical exercise behavior in depth [19, 20]. Studies have shown that physical health beliefs are the basis of exercise behavior and an essential link between health-related cognition and behavior [21]. That healthy behavior often needs to be supported by physical health beliefs [22]. Some researchers have demonstrated that exercise behavior is non-existent without positive exercise intentions [23], so it would appear that exercise intention is a necessary but insufficient predictor of exercise behavior. Consequently, concepts from a diverse range of behavioral theories, such as the health belief model [24] and the habit theory [25], have been utilized to add to the predictive validity and explanatory capacity of the theory of planned behavior in the exercise domain. These research efforts aim to refine the antecedents of movement intentions and behavior and identify variables that may facilitate the enactment of positive movement intentions. It is easy to see that health beliefs impact students in areas such as health perceptions and mental health and in predicting their behavioral intentions to exercise. Therefore, finding ways to promote healthy beliefs on health behaviors is essential. Consistent with the proposal, we formed our hypotheses 1 as follow: physical health beliefs significantly positively predicts physical exercise behavior intention of college students.

Research has found that high physical health beliefs do not necessarily promote physical exercise behavior intention [26]. The mechanism of physical health beliefs on physical exercise behavior intention, i.e., the process by which physical health beliefs impact physical exercise behavior intention, may have other mediating variables involved. Therefore, this study investigates this using a mediation model to explore the mechanism of action. Exercise imagery is widely used in sports training as a mental practice of action. Mental practice, when combined with physical practice, can be beneficial to beginners learning a sport. Imagery is a process that mobilizes all of the senses of the human body; it involves visualizing the physical properties of things, people, or places or recalling past experiences in mind and reshaping them [27]. Exercise imagery is the individual’s thoughts, emotions, or images that occur before the mental formation of the psychological image [28], and it can positively impact the implementation of the exercise [3]. In recent years, researchers have paid increasing attention to exercise imagery. Studies have shown that it is an essential factor in explaining the physical behavior of participants during physical exercise [29–31]. Man (2011) reported that exercise imagery positively affects cognition and motivation by raising awareness of motion [32]. Furthermore, Stanley’s (2010) research found that exercise imagery that involves a pleasant imagery of the exercise can produce goal-like results that play a positive role in physical exercise participation and its continuation [33]. It has been well-documented that imagery is effective in changing thoughts, beliefs, and behaviors in various domains [34, 35]. Additionally, imagery may be a self-regulatory strategy for exercisers to enhance motivation and self-efficacy [36]. Several studies have confirmed that imagery is used frequently by exercisers and is related to exercise cognition, such as exercise intentions [37], self-efficacy [38], and exercise motivation [39]. Researches found that exercise imagery that involves a pleasant image of the exercise can produce goal-like results that positively affect physical exercise participation and its continuation [40]. It has been well-documented that imagery effectively changes thoughts, beliefs, and behaviors in various domains [34, 41].

Exercise imagery as a perception of health beliefs gives individuals a sense of control and efficacy, which can enhance the motivational variable of exercise behavioral intention [42]. On the other hand, the four elements of cognition-emotion-behavioral intention-behavior are a recursive mechanism of action [43]. Empirical studies have also confirmed that exercise imagery can significantly and positively predict exercise behavioral intentions [44]. However, from examining the literature, it has been discovered that there needs to be more research and analysis on the influence of exercise imagery on the tendency to exercise and on the role of the mediator factors in China. Therefore, this study used a mediation model to continue this research in-depth. Based on the above discussion, by combining the aforementioned hypotheses, this study proposes the following hypotheses 2: exercise imagery play a mediating role between physical health beliefs and physical exercise behavior intention.

To sum up, this study aimed to explore the relationships between college students’ physical health beliefs, exercise imagery, and physical exercise behavior intention and to examine the mediated effect of exercise imagery in the relationship between physical health beliefs and physical exercise behavior intention. This study plans to build a mediating model. The in-depth discussion of the mechanism underlying the effect of physical health beliefs on physical exercise behavior intention will contribute to providing practical guidance and interventions for college physical exercise education.

## Materials and methods

### Subsection

From October to December 2022, an online survey system was used. To decrease the harmful effects of sampling bias, this study used a random sampling method. The Sojump website (https://www.wjx.cn/) was used to design the questionnaire and to generate a network link that was shared in classrooms at two universities via WeChat (Tencent Holdings Ltd., Shenzhen, China) or QQ group (Tencent Holdings Ltd., Shenzhen, China). The G*Power 3.1 program was used to determine the sample size and power. Results a minimum of 352 samples were reached for models with an alpha level of 0.05 and power level of 0.80. This many participants can be considered sufficient for the sample size in the current study [45].

The college students were invited to complete the questionnaire, and 1648 questionnaires were returned. The geographical analysis by Sojump indicated that 22 provinces and municipalities in Henan, Guangdong, Hebei, Jiangsu, Beijing, and Hubei, and other provinces and municipalities, completed the questionnaire. To improve the authenticity of the data, the actual completion time and quality of the online questionnaires were examined, and after the exclusion of invalid questionnaires(such as answer the questionnaire in less than 120s; and regular answers), there were 1356 remaining valid questionnaires (validity rate = 82.3%). The participants were aged from 17 to 23 years(Mage = 20.36 years, SD = 1.15). Of the respondents, 771 were male (56.9%) and 585 were female (43.1%), and 417 were freshman (30.8%), 449 (33.1%) were sophomores, 351 (25.9%) were juniors, 83 (6.1%) were seniors, and 56 (4.1%) were graduate students.

This study was approved by the Ethics Committee of School of Education at Zhengzhou University (ZDLL-2022135). All of the procedures were performed in accordance with the Declaration of Helsinki and relevant policies in China. All participants agreed to participate voluntarily, with informed consent when they fled in the survey and were able to withdraw from the study freely at any time. Questionnaire was designed and applied to ensure anonymity of participants. The data were confidential and participation was anonymous without any potential risk to the integrity of the subjects.

### Physical health beliefs scale for college students

The Physical Health Beliefs Scale for college students, which was compiled by Dai (2011), was employed. The scale consists of five dimensions and a total of 24 questions [46]. A 5-point scale was used, which ranged from 1 (complete non-conformity) to 5 (full compliance). A higher score indicates that the college students have a higher level of physical health beliefs. In this study, the fit indices of a confirmatory factor analysis’s model of the scale were as follows: root mean square error of approximation (RMSEA) = 0.07, comparative fit index (CFI) = 0.93, Tucker-Lewis index (TLI) = 0.90, and standardized root mean square residual (SRMR) = 0.06. In addition, the Cronbach’s alpha coefficient was 0.92. This indicates that the structure of the instrument is good.

### Exercise imagery

The Exercise Imagery, which was developed by Giacobbi and Hausenblas (2003), was used. This scale has been translated to Chinese, demonstrates adequate psychometric properties [47]. It consists of four dimensions and a total of 19 questions. A 5-point scale is used that ranges from 1 (rarely) to 5 (often); the higher the score, the higher the level of the college students’ exercise imagery. In this study, the fit indices of a confirmatory factor analysis’s model of the scale were RMSEA = 0.04, CFI = 0.91, TLI = 0.91, and SRMR = 0.05, and the Cronbach’s alpha coefficient was 0.90, which indicates that its structure is good.

### Exercise behavior intention scale

The physical exercise behavior intention subscale of the Exercise Attitude Scale (Zhang, 2004) was used [48]. The subscale has a total of eight questions, and it uses a 5-point scale that ranges from 1 (complete non-conformity) to 5 (full compliance). The higher the score, the higher the college students’ intention to exercise. In this study, the fit indices of a confirmatory factor analysis’s model of the scale were RMSEA = 0.05, CFI = 0.95, TLI = 0.94, and SRMR = 0.03, and the Cronbach’s alpha coefficient was 0.93, which demonstrates that its structure is good.

### Statistical analysis

This study has adopted IBM SPSS22.0 and Mplus8.1 statistical software for all data analyses. After the questionnaires were collected, all the data have been processed as follows: (1) Exploratory factor analysis was performed on all scales by SPSS22.0; (2) confirmatory factor analysis was performed on all scales by Mplus8.1; (3) internal consistency was tested for all scales by SPSS22.0; (4) the Harman single-factor method has been adopted for the common method deviation test; (5) descriptive statistics, such as statistical means (M), standard deviations (SD), maximum and minimum values, and the Cronbach’s alpha were computed; (6) Pearson correlation analysis to explore the relationship between physical health beliefs, exercise imagery, and physical exercise behavior intention; (7) T-tests were used to analyze whether there were gender differences in each variable; (8) a Structural Equation Modelling (SEM) approach was employed to test the theoretical model in the current study. PROCESS version 3.3 macro was used to construct the structural equations and to test the mediating effects [49]. The accepted level of significance was *p* < 0.05.

## Results

### Common method variance test

As all of the variables in this study were measured using the participants’ self-report, it was necessary to test their common method variance [50]. Harman’s single-factor method was used to verify if there was a common method bias. An exploratory factor analysis (without rotation) of all of the questionnaire items found that the first factor explained 26.4% of the variance, which is less than the critical standard of 40%. And using Mplus 8.1, all variables were set as 1 common factor and each item of all variables was used as an exogenous variable for CFA, and the results showed that:the fit indices of the model (χ2/df = 7.54, CFI = 0.649, TLI = 0.322, RMSEA = 0.091) were not satisfactory, so there was no serious common method bias [51].

### Correlations and descriptive statistics

The descriptive statistics and analyses of the variables are shown in Table 1. Physical health beliefs, meaning in life, exercise imagery, and physical exercise behavior intention were significantly positively correlated. An independent-samples t-test was used to test for gender differences in each variable, and it was found that the male students’ level of physical health beliefs and physical exercise behavior intention were significantly higher than that of the female students (*p* < 0.05, respectively). It was also found that the males’ exercise imagery scores was higher than the female students’ scores (*p* > 0.05), but there was no significant difference. As the males scored higher on these variables than the females, the follow-up analysis treated them as control variables to avoid the effects of gender and grade.

**Table 1:** Descriptive statistics and correlations (*N* = 1356)

Variables					Male		Female		t	*p*
	1	2	3	4	M	SD	M	SD		
1. PHB	-				4.14	0.50	3.91	0.63	2.75	0.03
2. EI	0.37**	0.25**			3.81	0.66	3.60	0.58	0.81	0.89
3. PEBI	0.31**	0.34**	0.28**	-	3.95	0.69	3.52	0.74	2.07	0.04

Note. PHB = physical health beliefs; EI = exercise imagery; PEBI = physical exercise behavior intention. **p < 0.01

### Mediation analysis

This study used the PROCESS macro Model 4 to test the mediation effects of exercise imagery. The results showed that physical health beliefs had a direct predictive effect on physical exercise behavior intention (β = 0.32, *p* < 0.001), This result confirms Hypothesis 1. In addition, exercise imagery had significant positive predictive effects on physical exercise behavior intention (β = 0.27, *p* < 0.001; see Table 2).

**Table 2:** Regression analysis of the mediation model

Regression equation		Overall fit		Regression coefficient significance	
Result variable	Predictors	R^2^	F	β	t
EI	Gender	0.24	26.71	-0.01	-0.31
	Grade			0.01	0.23
	PHB			0.48	13.46***
PEBI	Gender	0.52	89.44	-0.01	-0.29
	Grade			-0.19	-3.17
	PHB			0.32	9.54***
	EI			0.27	6.48***

Note. The variables in the model were standardized and entered into the regression equation. **p < 0.01, ***p < 0 0.001

**Fig. 1:**
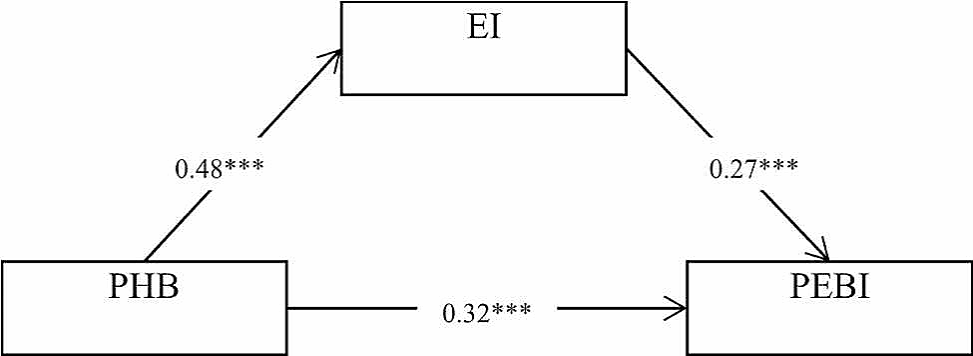
Diagram of structural equation model

The analysis of the mediation effects of exercise imagery showed that the mediation effect of exercise imagery was 0.13, and its bootstrap 95% confidence interval did not contain 0 (0.12, 0.36), which indicates that its mediation effect was significant (see Table 3; Fig. 1), This result confirms Hypothesis 2.

**Table 3:** Mediation effect analysis

	Indirect effects	SE	Bootstrap lower CI	Bootstrap upper CI	Relative mediation effect
Direct effect	0.32	0.03	0.12	0.26	71.12%
Mediating effect	0.13	0.01	0.08	0.17	28.89%
Total effects	0.45	0.04	0.17	0.38	

Note. SE = standard error; CI = confidence interval

## Discussion

Understanding college students’ physical health beliefs, exercise imagery, and physical exercise behavior intention during follow-up health education, and comprehending the level of their tendency to play sports by exploring and studying its influencing mechanism, is conducive to enhancing the transition from cognition to behavior. Based on the health belief model, cognitive-behavioral theory, and related research results, this study has analyzed and will discuss the relationship between college students’ physical health beliefs and physical exercise behavior intention and the underlying mechanism. This study found a significant positive correlation between physical health beliefs and physical exercise behavior intention. Further, when exercise imagery were entered into the mediation model, the results showed that meaning in life and exercise imagery perform a partial mediating function.

Given the positive consequences associated with physical exercise behavior intention, understanding the psychological and cognitive antecedents that underpin this construct is important. A relevant cognition associated with physical exercise behavior intention, also predicting exercise participation, is physical health beliefs [52]. Therefore, this study explored the effect of college students’ physical health beliefs on their physical exercise behavior intention and found that physical health beliefs significantly positively affect physical exercise behavior intention, which is consistent with the results of previous research [53]. Beliefs guide human action, guiding aspirations and determining people’s actions, and acquiring beliefs establish habits [54]. During the critical period of socialization and development of self-consciousness, adolescents’ awareness of the state of their bodies, physical activities, and mental activities has a profound impact on their personality development [55]. Studies have shown that when adolescents know the importance of physical fitness, they will tend to maintain good physical fitness and improve poor physical fitness, form their own rules of action based on their evaluations and the comparisons of others, and then form exercise behaviors [56].

The physical health belief model suggests that when an individual believes that his/her environment is a threat to his/her health, he/she will show a particular motivation to exercise and improve his/her health [57]. The results further show that the college students’ health awareness has improved and that they believe physical exercise is a way to stay healthy. According to cognitive-behavioral theory, individuals first produce a cognition (such as a physical exercise behavior intention), and this then influences their behavioral habits (i.e., physical exercise behavior) through behavioral effects (i.e., physical exercise behavior) [58]. At the same time, the peak-end rule argues that when people experience something, all they remember is the experience at the peak and the end [59, 60]. Recent studies have shown that groups with different health beliefs produce different health behaviors, and groups with the same health beliefs also have different health behaviors, which may be due to differences in individuals’ behavioral tendencies [61, 62]. Knowledge-Attitude/Belief-Practice(KAP) theory suggests that human behavior is related to cognition, values, and beliefs and that establishing health beliefs is a critical component in promoting physical exercise behavior [63]. The central aspect of college sports work is cultivating students’ sports literacy and lifelong consciousness of sports. Therefore, college sports work should constantly strengthen education about physical health beliefs because they directly affect physical exercise behavior intention and, in turn, influence the development of exercise behavior. Understanding college students’ physical exercise behavior intention and guiding them to participate in sports correctly requires further discussion in the field of physical education in colleges and universities.

A mediating variable is an internal factor that plays a role in the influence of physical health beliefs on physical exercise behavior intention. This study found that exercise imagery was mediating variable between physical health beliefs and physical exercise behavior intention. This study confirms that physical fitness beliefs do not just have a superficial and direct effect on college students’ propensity for physical activity. It is deeply embedded with a series of complex mediating links that differentiate the physical exercise behavior intention of college students with the same level of beliefs. Studies showed that exercise imagery may can increases self-determination and is the main reason for sustained sports participation [31]. In addition, exercise imagery can positively predict physical exercise behavior intention and positively impact the continuing intention to exercise [32]. Exercise imagery are cognitive mental imagery of thoughts, feelings, or images that an individual has about a physical exercise that is formed prior to physical exercise action and which can have a positive effect on physical exercise behavior intention [64]. Exercise imagery can further increase awareness of motor persistence. At the same time, Exercise imagery can vary with factors such as an individual’s past perceptions of exercise and physical exercise behavior intention. Exercise images can play a solid motivational role in exercise behavior, accompanied by the effects of self-efficacy and outcome expectancy, influencing exercise participants’ physical exercise behavior intention.

The findings showed that exercise imagery is an important intervention strategy for changing the psychological activities of physical exercise behavior intention, and that it can play a role in strengthening the mechanism to regulate and change physical exercise behavior. To improve college students’ physical exercise behavior, enhancing their exercise imagery level will be an effective intervention [65]. If college students do not actually exhibit sports behavior, they can form an abstract picture of physical exercise through exercise imagery, imagine the direct or indirect process of performing the physical exercise, and maintain a high level of expectation of the physical and psychological benefits of the physical exercise behavior to improve their physical exercise behavior intention more efficiently. This study emphasizes the importance of college students’ exercise imagery and provides a theoretical basis for follow-up research related to exercise imagery and physical exercise behavior. Therefore, when focusing on the relationship between physical health beliefs and physical exercise behavioral intentions of college students, it is also necessary to pay more attention to the “bridging” role played by exercise imagery.

### Practical implications

This study explored the influence of college students’ physical health beliefs on physical exercise behavior intention and further explored the intermediary roles of exercise imagery. The findings validate the cognitive evaluation model of physical health beliefs and the theoretical model of the sense of meaning in life. Exploring the relationship between physical health beliefs, exercise imagery, and physical exercise behavior intention can also provide a theoretical basis for how college sports work can promote college students’ sports behavior and cultivate their sports literacy and lifelong concept of sports.

### Limitations and future direction

Although this study has explored the internal mechanism of physical health beliefs affecting college students’ physical exercise behavior intention, since this study adopted self-reported report, there are the following problems: (1) All the subjects in this study are college students, and subsequent studies can investigate other groups to expand the sample representation; (2) This study is a correlation study in nature and cannot prove causality. Longitudinal tracking of the relationship between experimental intervention variables should be adopted in the future to improve the external validity of the study.

## Conclusions

We inferred in our study that physical health beliefs are of crucial importance for ensuring physical exercise behavior intention, while results shows the significance of physical health beliefs in fostering physical exercise behavior intention through exercise imagery. To conclude, (1) physical health beliefs can directly influence physical exercise behavioral intentions; (2) exercise imagery are mediators between between physical health beliefs and physical exercise behavioral intention among college students.

## Data Availability

The data presented in this study are available on request from the corresponding author.
